# Transient antibody-antigen interactions mediate the strain-specific recognition of a conserved malaria epitope

**DOI:** 10.1038/s42003-018-0063-1

**Published:** 2018-05-31

**Authors:** Bankala Krishnarjuna, Toshihiko Sugiki, Rodrigo A. V. Morales, Jeffrey Seow, Toshimichi Fujiwara, Karyn L. Wilde, Raymond S. Norton, Christopher A. MacRaild

**Affiliations:** 1 Medicinal Chemistry, Monash Institute of Pharmaceutical Sciences, Monash University, Parkville, VIC 3052 Australia; 2 Laboratory of Molecular Biophysics, Institute for Protein Research, Osaka University, Suita, Osaka 565-0871 Japan; 3 National Deuteration Facility, Australian Nuclear Science and Technology Organisation, Lucas Heights, Sydney, NSW 2234 Australia

## Abstract

Transient interactions in which binding partners retain substantial conformational disorder play an essential role in regulating biological networks, challenging the expectation that specificity demands structurally defined and unambiguous molecular interactions. The monoclonal antibody 6D8 recognises a completely conserved continuous nine-residue epitope within the intrinsically disordered malaria antigen, MSP2, yet it has different affinities for the two allelic forms of this antigen. NMR chemical shift perturbations, relaxation rates and paramagnetic relaxation enhancements reveal the presence of transient interactions involving polymorphic residues immediately C-terminal to the structurally defined epitope. A combination of these experimental data with molecular dynamics simulations shows clearly that the polymorphic C-terminal extension engages in multiple transient interactions distributed across much of the accessible antibody surface. These interactions are determined more by topographical features of the antibody surface than by sequence-specific interactions. Thus, specificity arises as a consequence of subtle differences in what are highly dynamic and essentially non-specific interactions.

## Abstract (web summary)

Krishnarjuna et al. show that multiple transient interactions mediate monoclonal antibody recognition of an epitope within a disordered malaria antigen, MSP2. These results explain the antibody’s differential affinities for two allelic forms of the antigen.

## Introduction

That a protein’s function and specificity is dictated by a well-defined three-dimensional structure has been a foundational assumption of molecular biology. However, the past two decades have seen the gradual recognition that the native states of many proteins include substantial regions that lack ordered structure^1^. These intrinsically disordered proteins are functional, not in spite of their disorder, but because their conformational flexibility affords functional possibilities not accessible to proteins with a more rigidly defined structure^2^. Disordered proteins often adopt a more defined conformation upon interaction with binding partners through a mechanism of coupled folding and binding^3^. However, it has been recognised recently that disorder can persist even in the presence of specific and high-affinity interactions, giving rise to interactions in which some or all of the interaction partners lack a well-defined conformation^4^. These disordered interactions are also called ‘fuzzy’ interactions, and are emerging as critical determinants of biomolecular specificity, particularly in the settings of cellular signalling and regulation, and in translational and transcriptional control^5^. These fuzzy interactions challenge the expectation that specificity demands well-defined and unambiguous molecular interactions, but the mechanisms by which specificity can arise from dynamic and ambiguous interactions remain unclear.

**Fig. 1:**
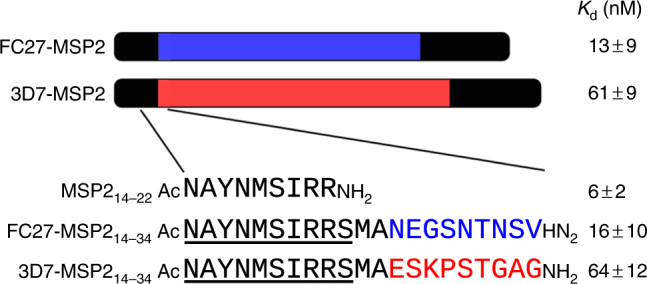
Schematic of the MSP2 constructs investigated here. Conserved regions are shown in black, and the largely dimorphic variable regions are red (3D7) and blue (FC27). The 6D8 epitope resolved by X-ray crystallography is underlined. Affinities for 6D8 scFv as previously determined by isothermal titration calorimetry are shown^6^.

Recently, we have identified fuzzy interactions in the unexpected context of an antibody–antigen interaction^6^. The malaria antigen merozoite surface protein 2 (MSP2) is an intrinsically disordered protein and is polymorphic, consisting of conserved N- and C-terminal regions and a central variable region containing dimorphic and polymorphic sequences^7, 8^. Based on the dimorphic sequences, MSP2 is classified into two allelic families, 3D7 and FC27 (Fig. 1)^8^. Antibodies targeting MSP2 arise in natural infections and in clinical trials of MSP2-based vaccines, and these antibodies contribute to clinical protection, albeit in a strain-specific manner^9^. The monoclonal antibody 6D8 binds a nine-residue linear epitope within the conserved N-terminal region of MSP2 with a *K*_d_ of 6 nM. This affinity is reduced by 2- and 10-fold, respectively, when binding full-length FC27 and 3D7 MSP2 (Fig. 1). These affinity differences are recapitulated by peptides that include the defined epitope and encompass as few as five residues from the variable regions of both MSP2 alleles^6^. This suggests that the variable-region residues adjacent to the defined epitope interact with the 6D8 antibody, giving rise to the observed difference in affinity. Nonetheless, these interactions were not resolved in crystal structures of 6D8 Fv with the extended epitopes 3D7 MSP2_14–30_ and FC27 MSP2_14–30_, and nuclear magnetic resonance (NMR) experiments indicated that the interactions are transient^6^. These transient interactions represent an unprecedented mode of antibody–antigen interaction, and may explain the difficulty in establishing broad neutralising responses to conserved disordered antigens.

Here we extend our studies of this system to characterise the interactions that mediate the strain-specific binding of 6D8 for the 3D7 and FC27 alleles of MSP2. We show that these interactions are broadly distributed over the accessible surface of the antibody and occur in the presence of persistent disorder in the interacting residues of MSP2. Moreover, we show that these interactions are remarkably insensitive to the variations in MSP2 sequence, depending instead on topographical features of the antibody surface. Thus, specificity arises in this system as a consequence of subtle differences in what are essentially non-specific interactions. Not only does this represent an unrecognised basis for specificity in an antibody–antigen interaction, it may also represent a general mechanism by which the interactions of disordered binding motifs are modulated by flanking sequences.

## Results

### Interactions are broadly distributed around the 6D8 paratope

Our initial NMR analysis of the fuzzy interactions between MSP2 and 6D8 was limited by the lack of resonance assignments for the 6D8 single-chain Fv (scFv)^6^. By combining partial deuteration of the 28 kDa scFv construct^10^ with structure-based assignment using resonance assignment by chemical shift prediction (RASP)^11^, we have now achieved complete backbone assignments for 210 of 218 non-proline residues, excluding both the (G_4_S)_3_ linker between the variable heavy chain (V_H_) and variable light chain (V_L_) domains, and the C-terminal His-tag. Extensive spectral perturbations on binding the peptide MSP2_14–22_ (Supplementary Fig. 1a) necessitated complete reassignment of this complex, which was achieved to a similar level of completeness.

**Fig. 2:**
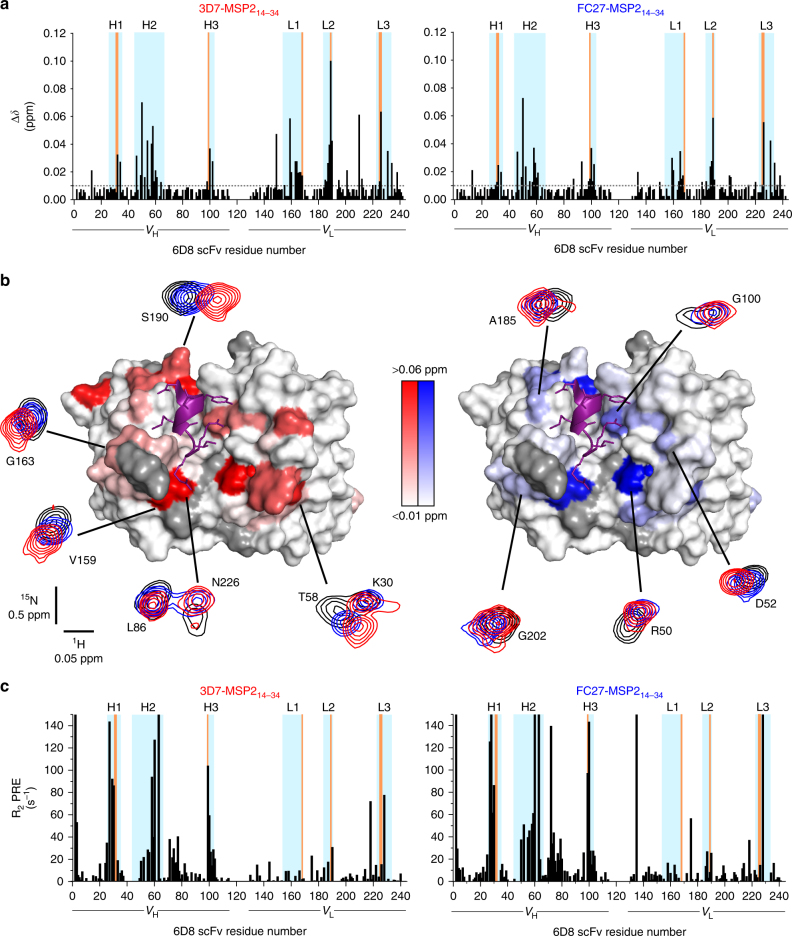
Chemical shift perturbations and PREs on 6D8 scFv reveal fuzzy interactions that mediate strain-specific recognition of MSP2. **a** Backbone amide chemical shift differences between 6D8 scFv in complex with MSP2_14–22_ and 3D7 MSP2_14–34_ (left) or FC27 MSP2_14–34_ (right). Regions corresponding to the 6D8 V_H_ and V_L_ are underlined, with CDRs^69^ highlighted in cyan and residues engaged in crystallographically resolved contacts with MSP2 in orange. **b** Backbone amide chemical shift differences span an extensive surface surrounding the structurally defined epitope (purple). Residues lacking data because of overlap or missing assignments are grey. Representative spectral differences are shown, with peaks shown from the [^15^N-^1^H]-TROSY of 6D8 scFv in complex with MSP2_14–22_ (black), 3D7 MSP2_14–34_ (red) and FC27 MSP2_14–34_ (blue). **c** PREs on 6D8 scFv arising from C-terminally MTSL-labelled 3D7 MSP2_14–34_ (left) or FC27 MSP2_14–34_ (right) show a similarly broad distribution

Comparison of the [^15^N-^1^H]-TROSY spectrum of 6D8 scFv in complex with MSP2_14–22_ with corresponding spectra of 6D8 scFv in complex with the two C-terminally extended epitopes, 3D7 and FC27 MSP2_14–34_, revealed only small differences, as observed previously^6^, which permitted straightforward transfer of assignments. Significant spectral perturbations were nonetheless observed, indicative of transient interactions of residues 23–34 of each MSP2_14–34_ peptide with 6D8 (Fig. 2a and Supplementary Fig. 1b; 36 residues show perturbations with *p* ≤ 0.05 for at least one peptide, after Bonferroni correction for multiple comparisons). Most of the perturbed resonances were from the complementarity determining regions (CDRs) of 6D8 scFv. However, there is only partial overlap between the perturbed resonances and those from residues directly involved in the crystallographically-defined interaction with MSP2_14–22_ (orange in Fig. 2a), emphasising that these perturbations reflect interactions that are not resolved in the crystal structure. Indeed, the perturbed resonances are distributed broadly across the extended paratope of 6D8, including all six CDRs as well as a few adjacent framework residues (Fig. 2b). The perturbations observed for the two extended epitopes, FC27 and 3D7 MSP2_14–34_, are highly similar, although some differences are apparent. They are often larger in magnitude for 3D7 than for FC27, although this is not universally the case (e.g., R50 and G202 in Fig. 2b). Likewise, they are generally but not universally collinear in the plane defined by the ^1^H and ^15^N chemical shifts (compare S190 and D52, Fig. 2b). It is difficult to account for these chemical shift differences in terms of a simple discrete interaction between 6D8 and the variable-region residues of the extended epitopes. Both the broad distribution of perturbations and the lack of consistent trends in their magnitude suggest a more diffuse set of competing interactions distributed across the surface formed by the 6D8 CDRs. Moreover, the similarity in the chemical shift perturbations induced by the two peptides implies that these interactions differ only subtly between FC27 and 3D7 MSP2.

To examine these interactions further, we attached the paramagnetic nitroxide probe *S*-(1-oxyl-2,2,5,5-tetramethyl-2,5-dihydro-1H-pyrrol-3-yl) methyl methanesulfonothioate (MTSL) at the C-terminus of the MSP2_14–34_ peptides, and measured the resulting distance-dependent paramagnetic relaxation enhancements (PREs) on the backbone amides of the bound 6D8 scFv. The distribution of PREs is broadly similar to that of the chemical shift perturbations (Fig. 2c), although with a clear bias towards the V_H_ domain of 6D8. As is the case with the chemical shift perturbations, the patterns of PREs seen for 3D7 and FC27 MSP2_14–34_ are similar, with only relatively minor differences in PRE magnitude seen between the two peptides (Supplementary Fig. 2). Indeed, after Bonferroni correction for multiple comparisons, none of the differences in PRE between 3D7 and FC27 MSP2_14–32_ approach statistical significance. Similarly, although 36 residues show significant chemical shift perturbations caused by either or both of the 3D7 or FC27 peptides, the difference in these perturbations is significant for only six residues.

**Fig. 3:**
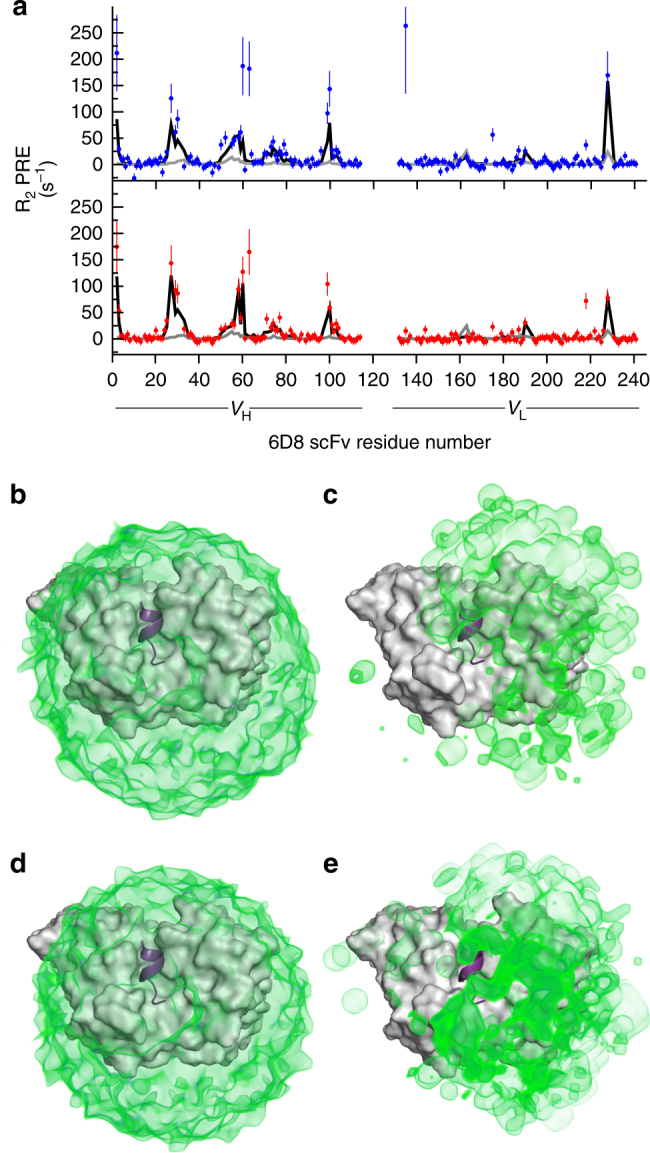
**a** PREs on 6D8 scFv arising from C-terminally MTSL-labelled 3D7 MSP2_14–34_ (top) or FC27 MSP2_14–34_ (bottom) are severely under-predicted by a Flexible-meccano-derived ensemble of MSP2_14–34_ conformations (grey line), but are adequately modelled after reweighting the ensemble under maximum-entropy constraint (black line). The unweighted ensemble results in a uniform and spherical distribution of probe positions for both 3D7 (**b**) and FC27 MSP2_14–34_ (**d**). The distribution arising from the reweighted ensembles (**c**, **e**) is less symmetrical, but retains a broad distribution across much of the accessible surface of 6D8, and is very similar between 3D7 (**c**) and FC27 (**e**)

We first sought to assess the extent to which the PREs report on fuzzy interactions involving the variable regions of MSP2. To this end, we used Flexible-meccano^12^ to generate conformational ensembles of either 3D7 or FC27 MSP2_14–34_ in complex with 6D8 scFv. In these ensembles the crystallographically resolved interactions are maintained, but the variable regions are modelled as disordered statistical coils, without additional interactions to the antibody. The PREs predicted by this model substantially underestimate the observed PREs (Fig. 3a, grey line), implying that Flexible-meccano predicts MSP2 peptide conformations that are, on average, further from the 6D8 scFv than is consistent with the PRE data. Thus, the PRE data offer further evidence for the extent of the transient interactions between the extended epitope and the antibody surface.

Attempts to model the experimental PREs in terms of a small number of discrete probe locations were also unsuccessful, with only modest agreement with the experimental PREs for as many as five independent probe positions (best fit *χ*^2^ > 2.3 for both FC27 and 3D7; Supplementary Fig. 3). This confirms the inference from the chemical shift perturbations that the conformations sampled by the MSP2 variable regions are diffuse and transient and are not dominated by a single (or even just a few) defined conformational states.

Importantly, however, agreement with the experimental data can be achieved by reweighting the relative populations of the members of the Flexible-meccano ensembles (Fig. 3a, black line), establishing that the PRE data are consistent with a model in which the variable regions of the extended epitope retain substantial disorder, but in which certain conformations are more strongly populated. This reweighting has the effect of increasing the magnitude of the predicted PREs to better fit the experimental data, implying that the contribution of conformers in which the paramagnetic probe is closer to 6D8 is emphasised in the reweighted ensemble. Indeed, the distribution of distances between the probe and the nearest 6D8 backbone amide is strongly skewed by the reweighting (Supplementary Fig. 4), resulting in a reduction of the average distance from approximately 38 Å in the two unweighted ensembles to 28 and 31 Å in the 3D7 and FC27 weighted ensembles, respectively. Similarly, the spatial distribution of probe positions is also strongly affected by the reweighting, shifting from the expected spherical distribution for the unweighted statistical coil ensemble (Fig. 3b, d), to an irregular distribution, with maximal density in a broad region covering CDR L3 and the three V_H_ CDRs of the 6D8 scFv (Fig. 3c, e). The breadth of probe positions sampled in the reweighted ensembles further emphasises that these data cannot be accounted for by a small number of discrete conformations of the variable C-terminal regions of the MSP2_14–34_ peptides. Rather, both the PRE and chemical shift data suggest that a wide range of conformations is sampled, with a diverse subset of these conformations being transiently stabilised by interactions with 6D8 scFv.

### Variable regions of MSP2 retain extensive disorder

**Fig. 4:**
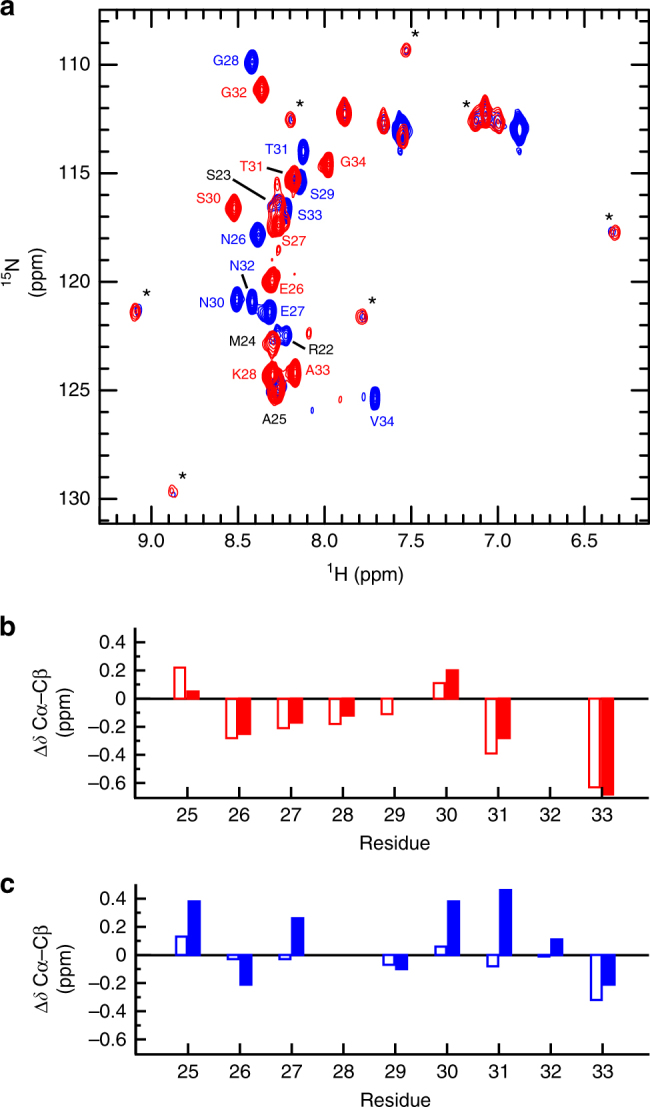
The conformational properties of the variable regions of MSP2_14–34_ are weakly perturbed by interaction with 6D8. **a** [^15^N-^1^H]-HSQC spectra of 3D7 MSP2_14–34_ (red) and FC27 MSP2_14–34_ (blue) in complex with 6D8 scFv. Assigned peaks are labelled and weak, unassigned peaks attributed to the structurally defined conserved residues are marked with asterisks. **b** Secondary Cα-Cβ chemical shifts for the variable residues of 3D7 MSP2_14–34_ free (open bars) and in complex with 6D8 scFv (closed bars). **c** Secondary Cα-Cβ chemical shifts for the variable residues of FC27 MSP2_14–34_ free (open bars) and in complex with 6D8 scFv (closed bars)

To directly test the model arising from the chemical shift perturbations and PRE data, we examined samples in which each extended epitope was uniformly ^13^C- and ^15^N-labelled and complexed with unlabelled 6D8 scFv. [^15^N-^1^H]-HSQC spectra of these samples revealed two classes of peaks. The first consists of intense peaks with limited spectral dispersion, suggesting conformational disorder, while the second class of peaks is much weaker in intensity but well dispersed, indicative of greater conformational order (Fig. 4a). The chemical shifts of the well-dispersed peaks were nearly identical for the 3D7 and FC27 epitopes, indicating that these peaks arise from the conserved and structurally defined epitope. In contrast, the chemical shifts of the more intense peaks differ between the two MSP2 peptides, but are similar to the peaks arising from the variable regions of the corresponding extended epitopes in the absence of 6D8 (Supplementary Fig. 5), establishing the assignment of these peaks. This similarity of chemical shifts implies that in spite of the complex interactions these variable-region residues make with the surface of 6D8, the average conformational properties of these regions are similar to those of free MSP2.

This view is further confirmed by ^13^C chemical shifts of the two sets of variable-region residues in complex with 6D8 scFv. These chemical shifts are similar to those expected for a disordered protein, and to those observed for the extended epitopes in the absence of 6D8 (Fig. 4b). Specifically, the secondary Cα-Cβ chemical shift difference is weakly negative for most of the variable-region residues of 3D7 MSP2_14–34_, both free (Fig. 4b, open bars) and in complex with 6D8 (closed bars). This implies a weak tendency to more extended conformations in this region^13^, which appears to be unaffected by the interactions with 6D8. In contrast, the secondary Cα-Cβ shift differences of the variable-region residues of FC27 MSP2_14–34_ are both positive and negative, and are slightly larger in the complex with 6D8 (Fig. 4c, closed bars) than they are for the free epitope (open bars), implying that the transient interactions with the antibody do alter the conformational properties of these residues, albeit weakly. Likewise, ^3^*J*_HN-HA_ scalar couplings for each peptide are close to those expected for a disordered protein, and those of the C-terminal half of the peptide do not change significantly on binding to 6D8 (Supplementary Fig. 6).

Attempts to assign the peaks corresponding to the conserved and structurally defined epitope were hampered by unfavourable relaxation properties and hence weak peak intensity, particularly in triple-resonance experiments. We have observed similarly unfavourable relaxation properties for other scFv-bound peptides^14^. For FC27, peaks corresponding to residues 22–25 could be assigned, whereas for 3D7, only residue 25 could be assigned. Peaks were identified in the [^15^N-^1^H]-HSQC spectrum of the 3D7 peptide with chemical shifts similar to those of FC27 residues 23 and 24 (Fig. 4a), but a lack of corresponding triple-resonance peaks precluded unambiguous assignment. Thus, the relaxation properties of these residues appear to differ between the 3D7 and FC27 extended epitopes, perhaps reflecting differences in the dynamics of these residues as a consequence of differing interactions with 6D8.

### Dynamic differences in the variable region of 6D8-bound MSP2

**Fig. 5:**
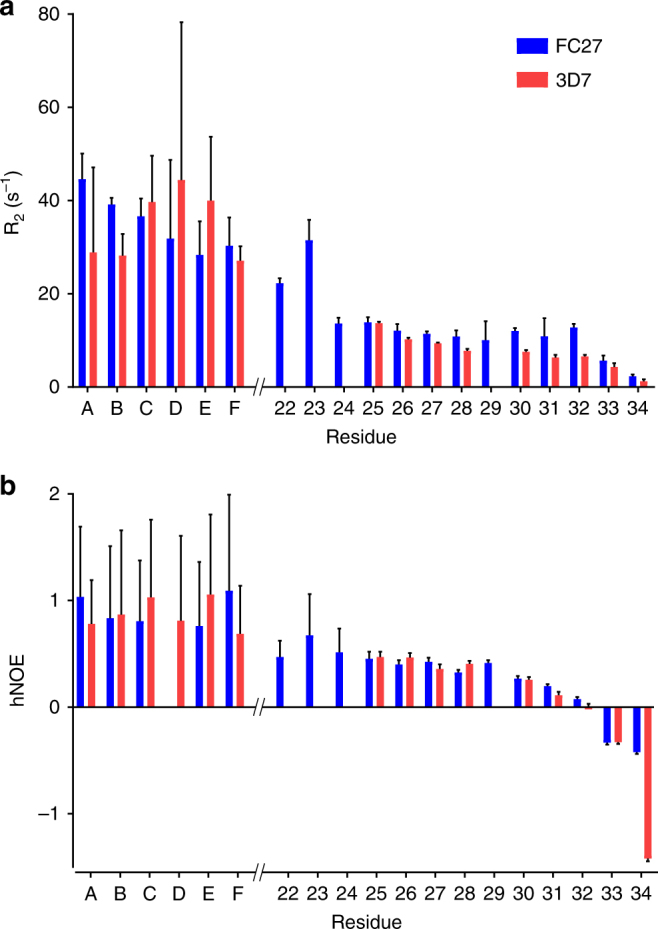
The dynamics of the variable regions of MSP2_14–34_ differ in complex with 6D8. ^15^N transverse relaxation rates (**a**) and {^1^H}^15^N hNOE (**b**) are lower for variable residues of 3D7 MSP2_14–34_ (red) than for FC27 MSP2_14–34_ (blue). Unassigned resonances arising from conserved regions of each peptide are labelled A–F. Error bars represent standard errors estimated from spectral noise

Backbone amide ^15^N transverse relaxation rates (*R*_2_) and {^1^H}^15^N heteronuclear Overhauser effects (hNOE) were measured to examine the dynamics of the 3D7 and the FC27 extended epitopes in the presence of 6D8 scFv. Although weak signals precluded the precise quantification of the relaxation properties of the conserved regions of the extended epitopes, they showed hNOEs approaching the maximum expected value and *R*_2_ > 30 s^−1^ (Fig. 5), consistent with a well-ordered conformation within the ~30 kDa complex with 6D8 scFv. In contrast, both *R*_2_ and hNOE values for the variable-region residues of the two extended epitopes were lower, further emphasising the persistent disorder across these regions. The observed relaxation parameters and their field dependence are consistent with conformational dynamics with a dominant timescale of approximately 1 ns over much of the variable region^15^. This is much faster than the overall rotational diffusion that dominates the relaxation of the scFv complex.

The variable regions of the two extended epitopes showed clear differences in their dynamics, suggesting some difference in their interactions with the 6D8 scFv. The *R*_2_ values for the 3D7 variable-region residues decreased monotonically towards the C-terminus, whereas the *R*_2_ values for the FC27 variable-region residues were higher than corresponding residues of 3D7 and were relatively uniform over residues 25–32 (Fig. 5a). This difference in *R*_2_ did not show a strong field dependence (compare Fig. 5a and Supplementary Fig. 7), implying that it arises from differences in ps-ns dynamics, rather than from slower exchange contributions to *R*_2_. Consistent with this interpretation, the hNOE values for FC27 were slightly higher than those for 3D7 over residues 30–34. These data indicate that the interactions between the MSP2 variable regions and 6D8 are highly dynamic on a ns timescale and reflect greater flexibility of the 3D7 MSP2_14–34_ variable region compared to the FC27 MSP2_14–34_ variable region when bound to 6D8 scFv. This observation is particularly striking in light of the proline residue at position 29 of 3D7 MSP2_14–34_, which, in the absence of other factors, would be expected to confer greater rigidity to the 3D7 variable region.

### Fuzzy interactions involve many short-lived interactions

The preceding analysis of NMR relaxation data implies that differences between the dynamics of the extended 3D7 and FC27 epitopes in complex with 6D8 occur on a timescale accessible to molecular dynamics (MD) simulation. Accordingly, we have used this approach to further explore the dynamic nature of the 6D8-MSP2_14–34_ complexes. In initial simulations using the Amber99SB-ILDN force field^16^, we observed interactions between the variable regions of both extended peptides that were stable on ~100 ns timescale, in apparent conflict with the NMR data, which suggest much more rapid exchange of these interactions. The appropriate choice of force field for the simulation of disordered proteins has been the subject of much recent work^17–21^, and several widely used force fields, including Amber99SB-ILDN, have been shown to overestimate the compactness and stability of interactions in disordered proteins^19, 21^. On the basis of these observations, it has been suggested that a force field that more appropriately balances protein–protein and protein–solvent interactions may be required to accurately model disordered proteins^22, 23^. In some of this work^19^, the CHARMM22^∗^ force field^24^ has been shown to yield more realistic conformational ensembles of disordered proteins^19^, prompting us to use it here. In simulations using this force field the variable regions of MSP2_14–34_ retain substantial flexibility while making transient interactions across a broad surface surrounding the 6D8 paratope, in qualitative agreement with our NMR data.

**Fig. 6:**
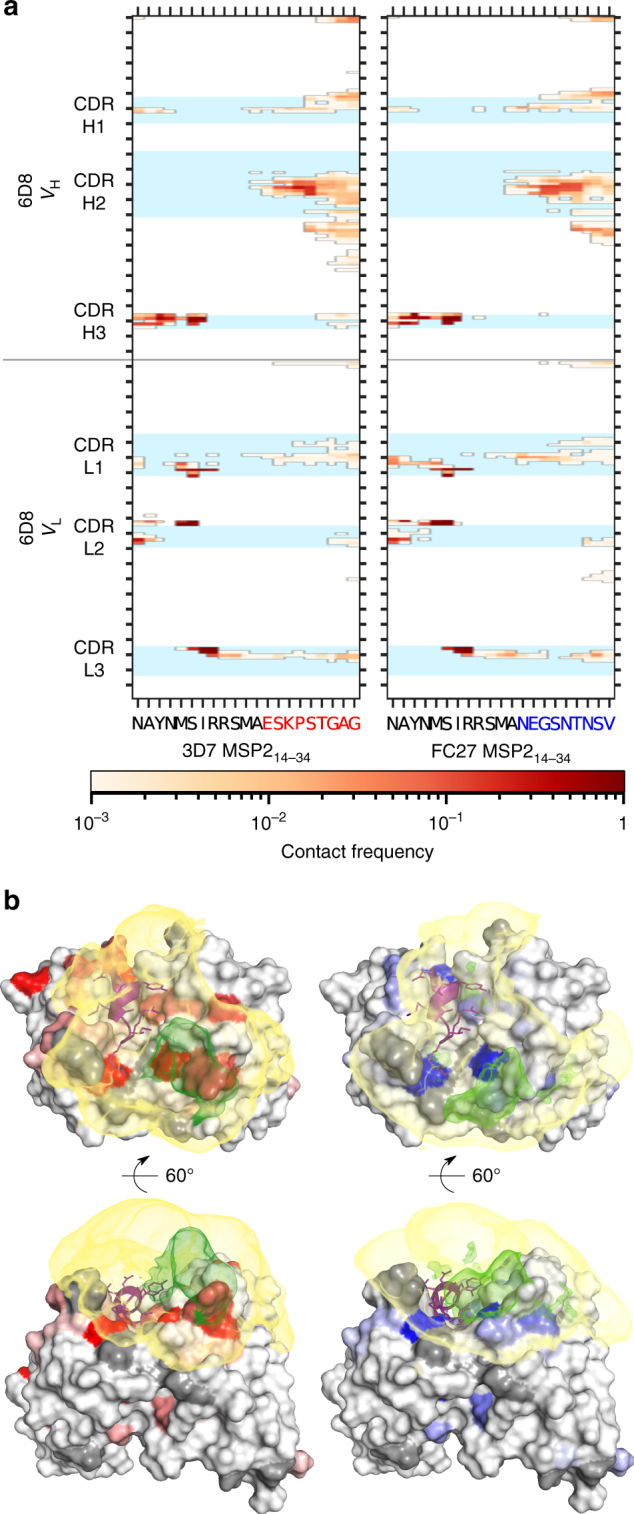
The fuzzy interactions made by the extended epitope variable regions are only weakly dependent on sequence. **a** The frequencies of residue contacts between 6D8 scFv and 3D7 MSP2_14–34_ (left) and FC27 MSP2_14–34_ (right) over ~1 µs of molecular dynamics simulation are remarkably similar. **b** The average density of MSP2 atoms involved in contacts with 6D8 is broadly distributed over the surface surrounding the 6D8 paratope for both 3D7 (left) and FC27 (right). Interacting atom density was calculated using MDAnalysis^68^ and rendered with PyMOL, contoured at 1.5 × 10^−3^ atoms per Å^3^ (yellow) and 20 × 10^−3^ atoms per Å^3^ (green). Chemical shift perturbations on 6D8 are shown as in Fig. 2, and the crystallographically resolved epitope (MSP2_14-22_) is shown in purple

To examine these interactions in more detail, we extracted residue contacts between 6D8 scFv and each of the extended epitopes from the MD simulations and mapped these contacts against the protein sequences (Fig. 6a). While the crystallographically resolved contacts are largely maintained across the full trajectory, numerous additional contacts are seen, involving the variable regions of the extended epitopes. The distributions of the observed interactions are similar in extent to the chemical shift perturbations seen on 6D8 scFv when the minimal epitope is extended (Fig. 6b). Consistent with the broad distribution of these variable-region interactions across the accessible surface of 6D8, they are significantly more numerous than those made by the structurally defined epitope: MSP2 residues 26–34 each make contacts with an average of 12.9 ± 2.6 residues on 6D8, whereas residues 14–25 each contact only 4.1 ± 0.9 residues. Likewise, the effective lifetimes of the interactions made by the variable region are shorter, with a median value of approximately 1 ns, and very few with lifetimes exceeding 10 ns, whereas many of the interactions involving the crystallographically defined epitope have lifetimes of 100 ns or more (Supplementary Fig. 8).

### Fuzzy interactions on 6D8 scFv are sequence-independent

Perhaps the most striking feature of the MD simulations is the similarity of the interactions made by the 3D7 and FC27 extended epitopes. There are only subtle differences in the locations, frequency and lifetime of the interactions involving the two variable regions (Fig. 6 and Supplementary Fig. 8). This result is consistent with the similarity of chemical shift perturbations and PREs in the complexes of both peptides (Fig. 2) and establishes that the interactions made by the variable regions of the two extended epitopes are relatively insensitive to the specific features of the sequences involved. Indeed, the distributions of the two sets of interactions on 6D8 scFv span most of the antibody surface within 20 Å of the end of the structurally-defined epitope, with variable-region residues of the two MSP2_14–34_ peptides making interactions with 29 (3D7) and 27 (FC27) of the 38 residues that comprise that surface. Moreover, these interactions appear to preferentially populate concave regions on the surface, with the distribution of interacting atoms of both extended epitopes appearing to be most dense in the otherwise unoccupied cleft between CDRs H2 and L1 (Fig. 6b, green density).

To explore this observation more quantitatively, we have used the PSP-score^25^, a measure of the extent to which a solvent-accessible point close to the 6D8 surface is enclosed within a surface concavity. The PSP-score was calculated over a 0.5 Å grid of all solvent-accessible points within 20 Å of the C-terminal end of the structurally-defined epitope and within 7 Å of the 6D8 scFv and compared with the density of interacting variable-region atoms mapped on the same grid. Grid points with a PSP-score ≥ 1 were considered to lie within a concavity on the antibody surface. Such points had a density of interacting atoms, averaged over the length of the MD simulation, of 4.2 × 10^−3^ atoms per Å^3^ (3D7) and 4.9 × 10^−3^ atoms per Å^3^ (FC27). This is approximately twice the interacting atom density of points with a PSP-score <1, for which the average values are 2.5 × 10^−3^ and 2.1 × 10^−3^ atoms per Å^3^, respectively. These results suggest that the interactions made by the variable regions of the two extended epitopes are determined largely by simple geometrical considerations, with the spatial range of the interactions limited by the reach of the disordered chain, albeit with a preference for concave regions of the antibody surface, in which denser and more efficient interactions are possible^26^.

## Discussion

The monoclonal antibody 6D8 recognises a conserved epitope within the N-terminal region of MSP2, yet it recognises the two allelic forms of MSP2, 3D7 and FC27, with five-fold difference in affinity^6^. We have previously established that the conformational and dynamic properties of the conserved regions of 3D7 and FC27 MSP2 are identical^27^, implying that additional interactions between 6D8 and the variable regions of MSP2 are responsible for the unexpected strain specificity of the antibody^6^. Our experimental and computational results establish that the variable-region residues of 3D7 and FC27 MSP2_14–34_ indeed make interactions with 6D8, but that these interactions are highly transient and are distributed over a broad region of the 6D8 scFv. In spite of these interactions, these variable-region residues retain substantial disorder, and this disorder allows them to sample a large fraction of the accessible antibody surface. The overall pattern of these interactions is remarkably similar between the two allelic forms of MSP2, with chemical shift perturbations, PREs and the MD simulations showing only minor differences between the two extended epitopes. Thus, sequence-specific interactions appear to play a relatively minor role in determining the extent, nature and timescale of these interactions.

Nonetheless, some sequence specificity must persist in order to account for the difference in affinity with which 6D8 recognises 3D7 and FC27 MSP2 (Fig. 1)^6^. Indeed, these differences are reflected in the distinct relaxation behaviour (Fig. 5) and secondary chemical shift changes (Fig. 4) seen for the variable regions of the two peptides. The simplest explanation for these differences is that the FC27 MSP2_14–34_ variable region interacts slightly more stably with 6D8 than does the corresponding region of 3D7 MSP2_14–34_, affording slower overall conformational dynamics, as inferred from the relaxation data, and slightly greater perturbation of the peptide conformation, as suggested by the chemical shift changes. This difference in affinity of the transient interactions of the variable regions of FC27 and 3D7 MSP2_14–34_ presumably gives rise to the difference in overall affinity of 6D8 for full-length FC27 and 3D7 MSP2.

The mechanisms by which transient interactions give rise to specificity in biomolecular interactions remain largely unclear, despite the rapidly increasing recognition of their importance. The results described here show that the transient interactions that give rise to strain-specific recognition of MSP2 by 6D8 are remarkably similar in the two allelic forms of MSP2, despite marked divergence in the sequences involved. The effective concentration of the MSP2 variable-region residues responsible for these interactions is very high, by virtue of their proximity to the structurally defined epitope that binds 6D8 with high affinity. Thus, these interactions can perhaps best be described as non-specific interactions that arise as a direct result of this high effective concentration. Similarly, high effective concentrations are inevitable at the flanks of a defined binding site within any disordered protein, implying that interactions of the sort characterised here are likely to be quite general.

Indeed, a broad class of fuzzy interactions is characterised by relatively weak sequence constraints, similar to those we observe here^28^. Perhaps the prototypical example of this class of interaction is the interaction between the Mediator subunit Med15 and the transcription activator Gcn4^29^. The disordered activation domain of Gcn4 binds any of at least three activator binding domains in Med15 with low affinity, and without a well-defined mode of interaction. Extensive mutagenesis of the activation domain establishes that there are few important sequence determinants of these interactions. There are also a number of well-characterised instances in which the affinity of disordered proteins for their binding partner is modulated by fuzzy interactions flanking the structurally defined binding motif^28, 30–33^. In light of the tendency for interaction motifs within disordered proteins to be flanked by regions rich in post-translational modification^34^, alternative splicing^35^ and sequence variation^36^, interactions involving these flanks potentially represent an important mechanism for both regulatory and evolutionary fine-tuning of interaction networks involving disordered proteins. Our findings suggest a mechanism by which such fine-tuning can occur in the absence of structurally well-defined interactions, and even in the apparent absence of sequence specificity.

Another interesting parallel was described very recently in the two-domain enzyme quiescin sulfhydryl oxidase from the parasite *Trypanosoma brucei*^37^. Here the two domains are joined by a short disordered linker, and the enzyme transitions between open and closed states in the course of the catalytic cycle. Fuzzy and apparently non-specific interactions between the two domains, arising as a result of their high mutual effective concentration, give rise to a rugged energy landscape, sub-diffusive conformational dynamics and ultimately to unusual, non-exponential reaction kinetics in this enzyme^37^. Thus, non-specific interactions, occurring as a result of the high effective concentration ensured by tethering with a disordered linker, can modulate protein function in a range of ways.

Intrinsically disordered proteins are frequent targets of antibody recognition in malaria^38^ and in a range of other infectious diseases^26^, and a number of both new and established vaccine candidates are disordered proteins^39–46^. As for disordered proteins more generally, disordered antigens are frequently polymorphic, and many evolve under selective pressure from the host immune system. Indeed, it is often argued that disorder is itself an adaptive response to the host immune system^47–49^, although this has been challenged on a number of grounds^26, 50^. Nonetheless, the observation that antibody recognition of a conserved epitope can be rendered strain-specific by transient interactions involving flanking variable sequence suggests a previously unrecognised mechanism by which disordered epitopes may evade immune surveillance.

## Methods

### Protein expression

The 6D8 scFv was ^2^H-,^13^C- and ^15^N-labelled by expression in *Escherichia coli* (*E. coli*) grown in media containing ^2^H_2_O, ^15^NH_4_Cl and ^13^C glycerol^10^, and purified as previously described^6^. Briefly, periplasmic contents were released by osmotic shock into 10 µg mL^−1^ DNAse in 2 mM MgCl_2_, and correctly folded 6D8 scFv isolated on MSP2 immobilised on *N*-hydroxysuccinimidyl-Sepharose beads. 6D8 scFv was eluted in 10 mL 0.1 M glycine, pH 2.5 and immediately neutralised by the addition of 2 mL of 1 M Tris, pH 8.0. Monomeric 6D8 scFv or its complex with MSP2 peptides was isolated by size-exclusion chromatography on Superdex 75 in 20 mM citrate and 50 mM NaCl, pH 6.5.

Genes encoding FC27 and 3D7 MSP2_14–34_ were obtained from GenScript and sub-cloned into pET32a vector for expression in *E.coli* BL21 strain. ^13^C- and ^15^N-labelled peptides were expressed and purified using an established protocol^27^.

### NMR spectroscopy

Backbone assignments for 6D8 scFv in the presence and absence of MSP2_14–22_ were determined from conventional triple-resonance experiments (Table 1) using CcpNmr Analysis 2.4.2^51, 52^ together with RASP^11^. Chemical shift perturbations were determined from two-dimensional [^15^N-^1^H]-TROSY spectra of 6D8 scFv recorded with 128 scans and 256 increments in the presence and absence of MSP2_14–22_, FC27 MSP2_14–34_ and 3D7 MSP2_14–34_. The concentrations of 6D8 scFv and peptides were 30 and 36 µM, respectively. The buffer used in these experiments contained 20 mM sodium citrate, pH 6.5, and 7% (v/v) ^2^H_2_O. Weighting of chemical shift perturbations from ^1^H and ^15^N resonances was performed using the following equation^53, 54^.

1 $${\mathrm{Weighted}}\,{\mathrm{CSP}}\,\left( {{\mathrm{\Delta}} {{\delta }}} \right) = \sqrt {\frac{1}{2}\left[ {{\delta }_{\mathrm{H}}^2 + \left( {0.14 \cdot {\delta}_{\mathrm{N}}} \right)^2} \right]}$$

where, *δ*_H_ and *δ*_N_ are the chemical shift perturbations in the ^1^H and ^15^N dimensions, respectively. The precision of Δ*δ* was estimated for each residue separately, by comparison of peak positions in three spectra of the 6D8 scFv:MSP2_14–22_ complex.

**Table 1:** Acquisition parameters for triple-resonance NMR experiments

Experiment	HNCO	HNCA	HN(CO)CA	HNCACB	HN(CO)CACB	HN(CA)CB
No. of scans	24	64	64	64	56	48
No. of data points						
F3 (^1^H)	2048	2048	2048	2048	2048	2048
F1 (^13^C)	42	64	128	128	128	128
F2 (^15^N)	128	42	42	42	42	42

Relaxation measurements for ^13^C- and ^15^N-labelled FC27 and 3D7 MSP2_14–34_ epitopes were made in the presence of a small excess of unlabelled 6D8 scFv on 600 and 950 MHz Bruker Avance III NMR spectrometers. In order to measure PREs, FC27 or 3D7 MSP2_14–34_ with a cysteine residue introduced at the C terminus of each peptide were labelled with an eightfold excess of *S*-(1-oxyl-2,2,5,5-tetramethyl-2,5-dihydro-1H-pyrrol-3-yl)methyl (MTSL) in 20 mM Tris, pH 8.0. The reaction proceeded in the dark under N_2_ at room temperature for 6 h before the spin-labelled peptides were purified using reversed-phase high-performance liquid chromatography. The purity and identity of the modified peptides were confirmed by liquid chromatography-mass spectrometry. Samples for PRE measurements were prepared by dissolving lyophilised 6D8 scFv and a small excess of the MTSL-labelled MSP2_14–34_ peptides in 20 mM sodium citrate buffer before isolation of the complex by size-exclusion chromatography. ^2^H_2_O was added to 7% (v/v) and the final pH was adjusted to 6.5. [^15^N-^1^H]-TROSY spectra with a recycle delay of 4 s, 24 scans and 256 increments were recorded before and after reduction of the nitroxide by addition of 250 µM sodium ascorbate. Peak intensities were determined by fitting in CcpNmr Analysis 2.4.2^51^, and PREs were determined from the ratio of reduced and oxidised peak intensities as described^55^.

PREs were modelled in terms of discrete probe locations using the structure of 6D8 scFv bound to MSP2_14–22_^6^ (PDB ID 4QXT) using the Gillespie-Shortle approximation^56, 57^ assuming an effective correlation time of 15 ns, corresponding to the overall rotational diffusion 6D8 scFv. Quality of fit was not significantly altered by choosing shorter values of the correlation time. Up to five equipopulated probe positions were selected using the basinhopping algorithm implemented in SciPy^58^, so as to optimise agreement between the experimental PREs and the average of the PREs calculated for each probe position. Each fit was repeated at least five times with random starting probe positions, and the fit with lowest *χ*^2^ selected.

An alternative model based on Flexible-meccano-derived ensembles of the 6D8 complex with MSP2_14–34_ was constructed as follows: 10 000 backbone conformations of each of 3D7 MSP2_14–34_ and FC27 MSP2_14–34_ were generated using Flexible-meccano^12^ with the conformation of residues 14–22 fixed in the crystallographically defined structure^6^. Each conformation was aligned with MSP2_14–22_ in 4QXT using the PDB.Superimposer module of Biopython^59^, to generate an ensemble of models of the complex with 6D8 scFv. To avoid steric clashes between MSP2 and 6D8, individual models in which Cα atoms of residues 23–34 of MSP2 were within 3 Å of any atom of the 6D8 scFv were discarded. This procedure resulted in ensembles of 7550 and 7421 conformers of the complexes of 6D8 with 3D7 and FC27 MSP2_14–34_, respectively. PREs were calculated for each ensemble member using the XPLOR-NIH v2.45^60^ implementation of the SBMF formalism^61^, using standard scripts distributed with XPLOR-NIH. Briefly, for each member of the two ensembles, five random conformations for the C-terminal cysteinyl-MTSL residue were generated using a simulated annealing protocol in which the positions of all other atoms were fixed. PREs were calculated for each ensemble assuming *τ*_c_ = 15 ns and *τ*_i_ ≪ *τ*_c_. Ensembles were reweighted to fit the experimental PREs under maximum-entropy constraint using a strategy essentially identical to COPER^62^, implemented with SciPy^58^.

### MD simulations

Initial models of the 6D8-MSP2_14–34_ complexes were constructed from the 6D8-MSP2_14–22_ crystal structure^6^ (PDB ID 4QXT), with the additional MSP2 residues initially built in an extended conformation. Simulations were run in GROMACS v5.1.3^63^ using either the Amber99SB-ILDN^16^ or CHARMM22^∗^ force field^24^. Each starting conformation was prepared for each force field separately, as follows: each system was subjected to 1000 steps of steepest-descent energy minimisation in vacuo, then solvated in TIP3P water^64^ containing 150 mM NaCl in a rhombic dodecahedron of 766 nm^3^ and subjected to a further 1000 steps of energy minimisation. Each system was thermalised by assigning velocities from a Maxwell distribution at 300 K, and dynamics was run for 0.1 ns in the NVT ensemble followed by 0.1 ns in the NPT ensemble, with positional restraints on all protein heavy atoms of 1000 kJ mol^−1^ nm^−2^. Finally, the extended conformation of each MSP2 variable region was relaxed with at least 20 ns of unconstrained dynamics at 300 K, followed by a total of 1 µs of production simulation for each complex. Production dynamics was run with periodic boundary conditions, using the LINCS algorithm^65^ to constrain bond lengths of H atoms, and a time step of 2 fs. Trajectories were stored with a time resolution of 2 ps. Cutoffs for non-bonded and short-ranged electrostatic interactions were 1.0 nm, and particle-mesh Ewald summation was used for long-ranged electrostatics^66^. Temperature was controlled with independent Nose-Hoover thermostats for protein and solvent, and pressure with the Parrinello-Rahman barostat.

Residue contacts are considered to exist for any pair of residues for which the Cα-Cα distance is <7 Å for more than 0.1% of the simulation length. Effective contact lifetimes are estimated by way of an autocorrelation function analogous to that defined for hydrogen bonds by Gowers and Carbone^67^:

$$C_{ij}\left( t \right) = \frac{{\mathop {\sum }\nolimits_{t_0} \delta _{ij}\left( {t_0} \right)\delta _{ij}\left( {t_0 + t} \right)}}{{\mathop {\sum }\nolimits_{t_0} \delta _{ij}\left( {t_0} \right)}}$$

where:

$$\delta _{ij} = \left\{ {\begin{array}{*{20}{c}} {1\,if\,d_{{\mathrm{C\alpha}} } \,\,< \,\,7{\AA}} \\ {0\,if\,d_{{\mathrm{C\alpha}} } \ge 7{\AA}} \end{array}} \right.$$

Effective lifetimes are taken as the time integral of a multi-exponential fit to *C*_*ij*_(*t*).

The average density of MSP2 atoms within 7 Å of any atom in 6D8 scFv was calculated over each trajectory on a 0.5 Å grid using MDAnalysis^68^. PSP scores were calculated on the same grid, as described^25^.

### Code availability

All custom code used in the data analysis is available at https://github.com/macraild/6D8-CommsBiol.

### Data availability

Assigned chemical shifts have been submitted to the BMRB with accession numbers 27222 and 27223.

## Electronic supplementary material

Supplementary Information
