# Supplementary material for: Transient antibody-antigen interactions mediate the strain-specific recognition of a conserved malaria epitope
Source: Commun Biol. 2018 May 31;1:58. doi: 10.1038/s42003-018-0063-1 (PMC6123721; doi:10.1038/s42003-018-0063-1)
Supplement: Supplementary file 1 — Supplementary Information [file 42003_2018_63_MOESM1_ESM.pdf]

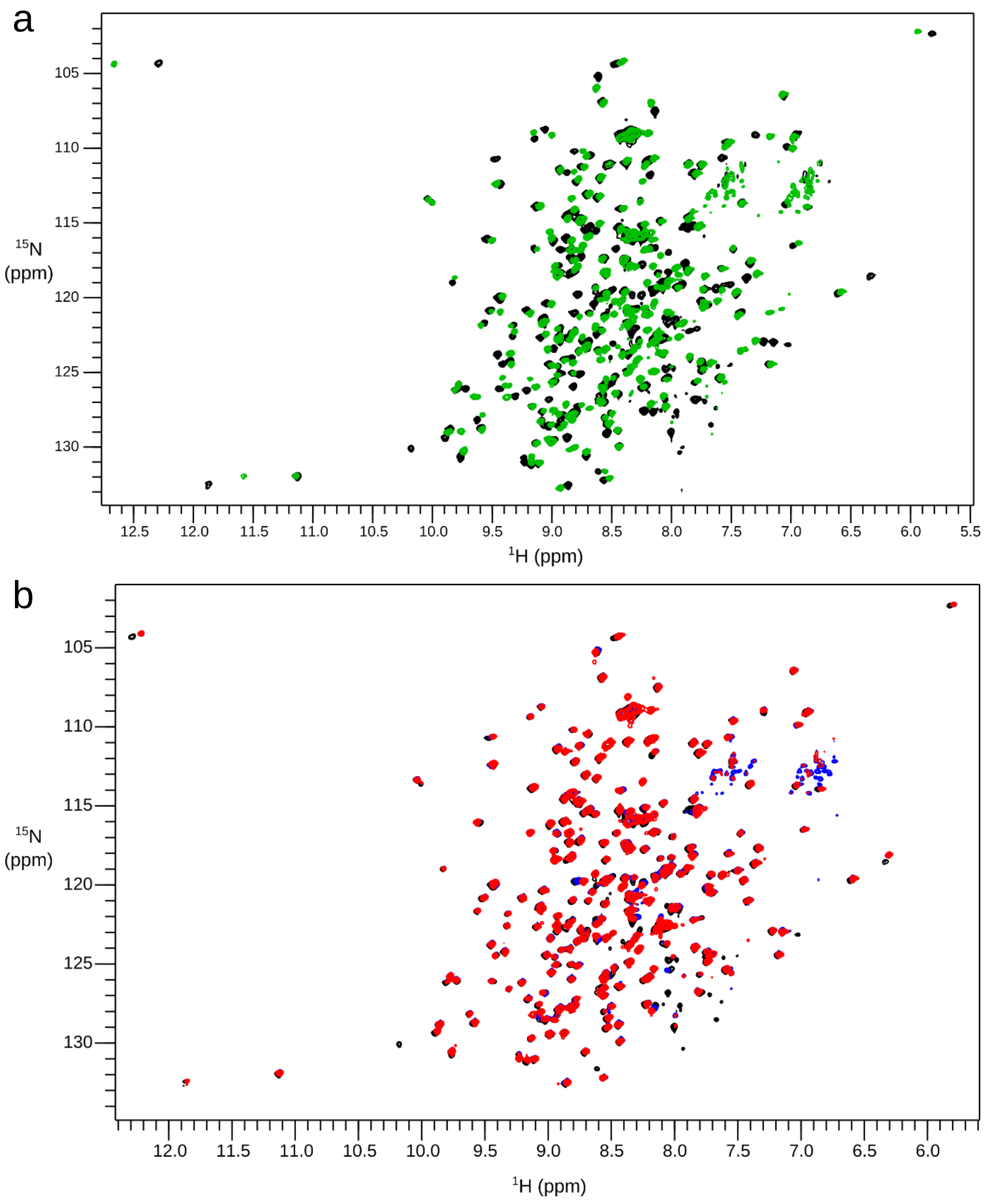

Supplementary Figure 1. [^{15}N-^{1}H]-TROSY spectra of triple-labelled 6D8 scFv (a) in the absence (green) and presence (black) of MSP2$_{14-22}$ and (b) in the presence of MSP2$_{14-22}$ (black), 3D7 MSP2$_{14-34}$ (red) and FC27 MSP2$_{14-34}$ (blue).

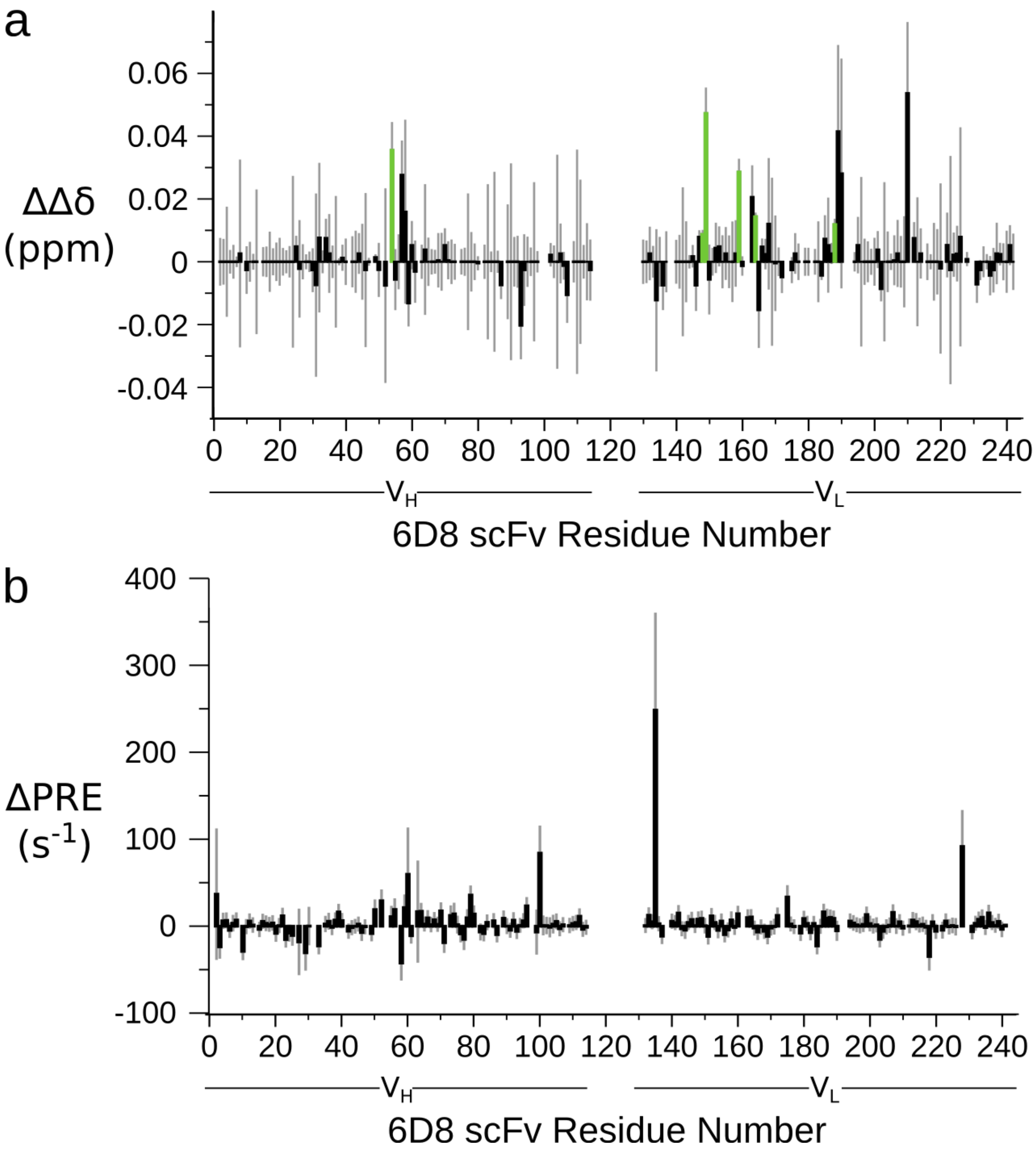

Supplementary Figure 2. Chemical shift perturbations and PREs reporting on the fuzzy interactions between MSP2$_{14-34}$ and 6D8 scFv are similar between 3D7 MSP2 and FC27 MSP2. (a) Differences between the chemical shift perturbations (ΔΔδ) on 6D8 scFv when binding 3D7 MSP2$_{14-34}$ *versus* FC27 MSP2$_{14-34}$ are plotted, with error bars estimated from repeat [^{15}N-^{1}H]-TROSY spectra recorded from a single sample. Statistically significant differences after Bonferroni correction are highlighted in green. (b) Differences in the PRE on 6D8 scFv bound to C-terminally MTSL-labelled 3D7 MSP2$_{14-34}$ *versus* FC27 MSP2$_{14-34}$ are plotted with error bars estimated on the basis of noise levels in the PRE experiments.

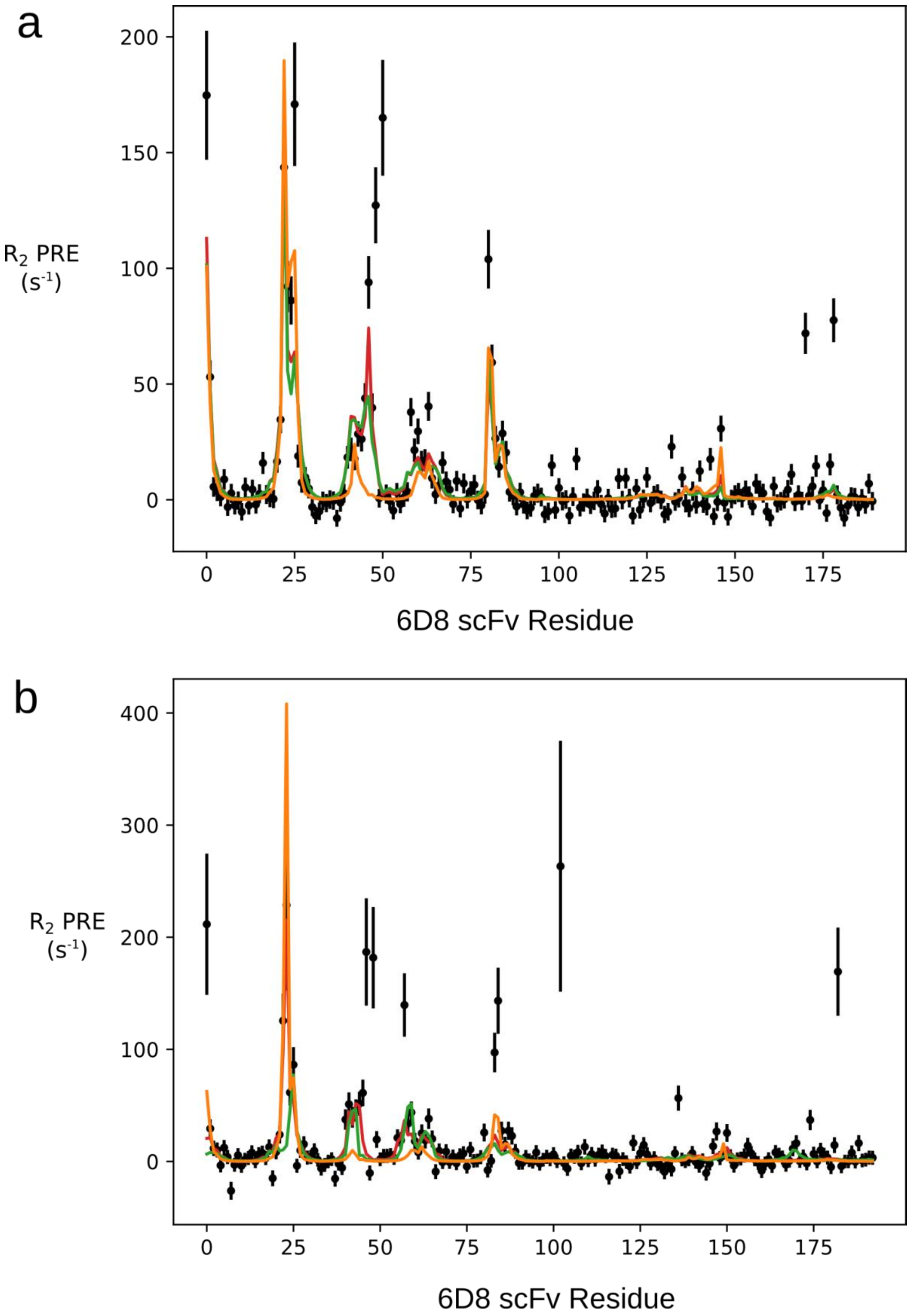

Supplementary Figure 3. PREs (black) measured on 6D8 scFv bound to 3D7 $MSP2_{14\text{-}34}$-MTSL (a) or FC27 $MSP2_{14\text{-}34}$-MTSL (b) and modelled on the basis of one (yellow), two (green), or three (red) discrete equipopulated nitroxide probe locations, as described in the main text.

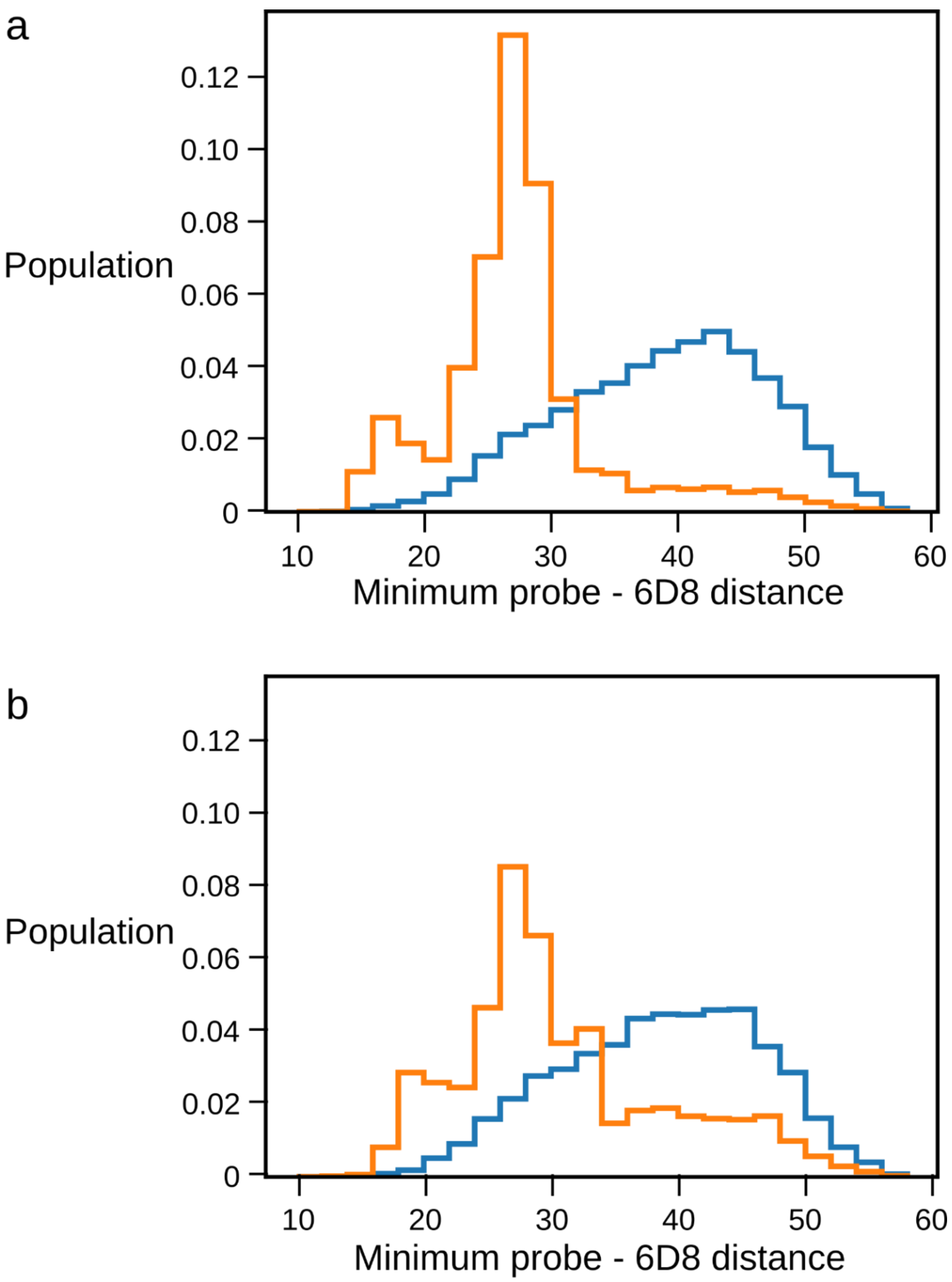

Supplementary Figure 4. Reweighting of the Flexible-Meccano ensembles of $MSP2_{14\text{-}34}$ conformations to fit the experimental PREs favours probe locations closer to 6D8. The distribution of distances from each probe to the nearest 6D8 scFv amide is plotted for the unweighted (blue) and weighted (orange) ensembles for 3D7 (a) and FC27 (b) $MSP2_{14\text{-}34}$. The difference in the extent of rewieghting can be attributed to differences in the precision of the two PRE datasets.

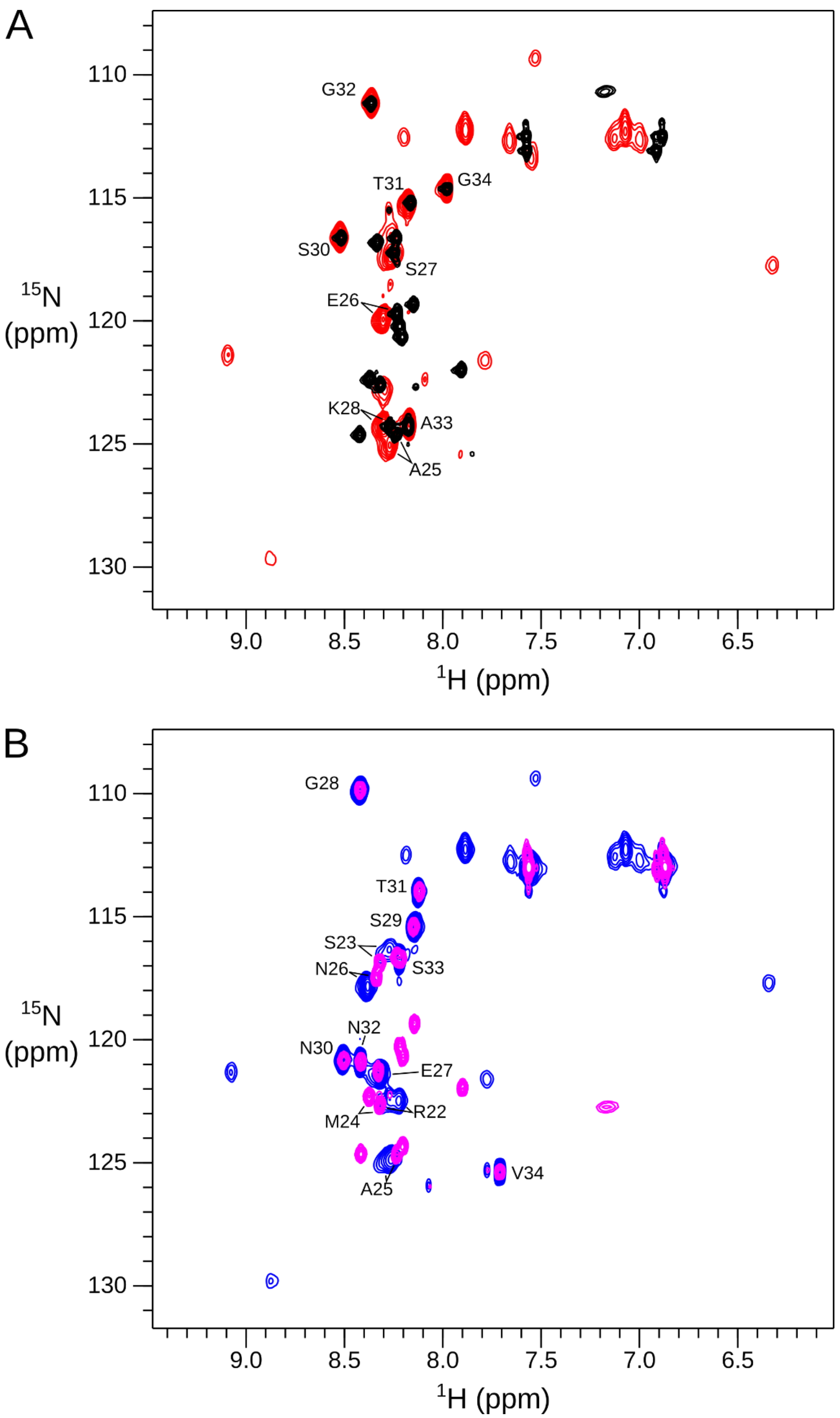

Supplementary Figure 5. (A) ^{1}H,^{15}N TROSY spectra of 3D7 $MSP2_{14\text{-}34}$ free (black) and bound to 6D8 scFv (red). (B) ^{1}H,^{15}N TROSY spectra of FC27 $MSP2_{14\text{-}34}$ free (magenta) and bound to 6D8 scFv (blue). Resonance assignments for the C-terminal half of each peptide are shown.

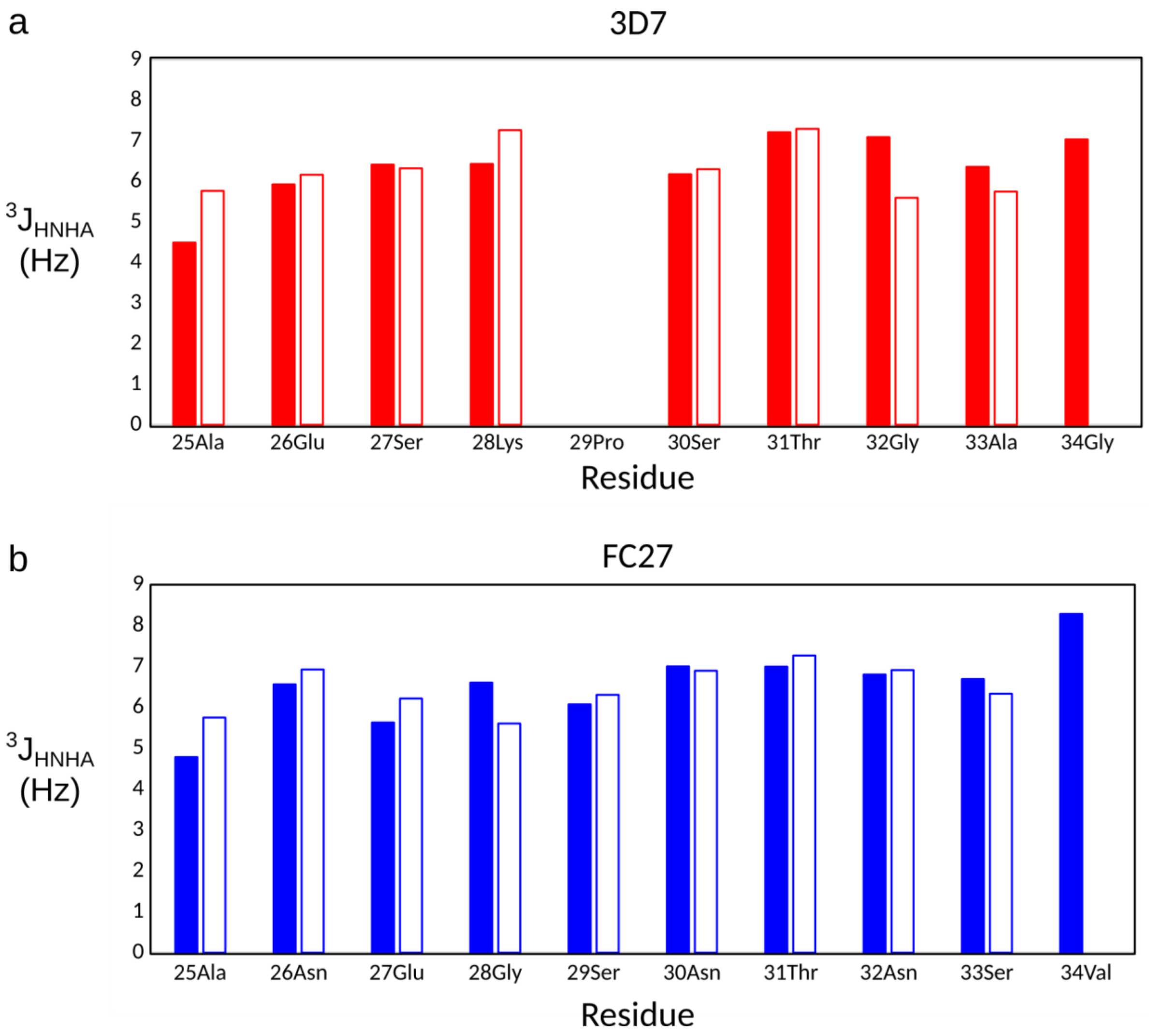

Supplementary Figure 6. $^3J_{\text{HN-HA}}$ scalar couplings of the variable-region residues of 3D7 $MSP2_{14\text{-}34}$ (a) and FC27 $MSP2_{14\text{-}34}$ (b) bound to 6D8 scFv (solid bars) are similar to each other and to $^3J_{\text{HN-HA}}$ couplings in the free peptide (open bars).

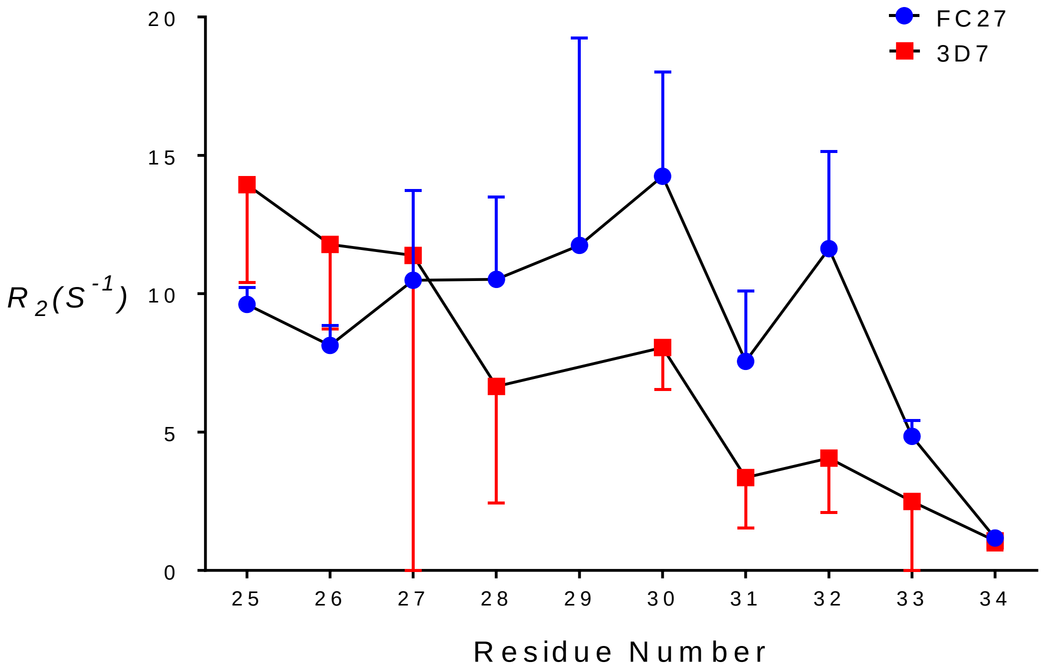

Supplementary Figure 7. Transverse relaxation rates (*R_2*) of 3D7 $MSP2_{14\text{-}34}$ (red) are lower than FC27 $MSP2_{14\text{-}34}$ (blue) for most variable-region residues at a ^{1}H Larmor frequency of 600 MHz.

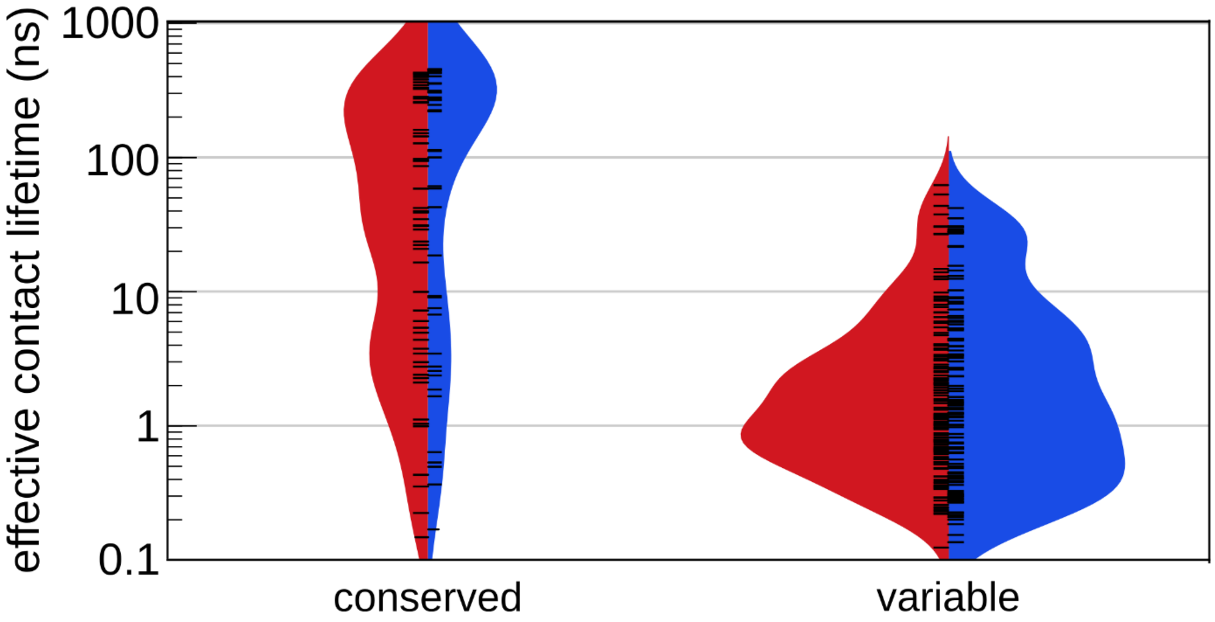

Supplementary Figure 8. The effective lifetimes of intermolecular residue contacts are longer for contacts involving the conserved region of the extended epitopes (residues 14-25, left) than for residues in the variable region (residues 26-34, right). Violin plots show the estimated distribution of lifetimes for 3D7 (red) and FC27 (blue). Individual residue contact lifetimes are shown as black ticks.
